# Aberrant Functional Connectivity of Resting State Networks in Transient Ischemic Attack

**DOI:** 10.1371/journal.pone.0071009

**Published:** 2013-08-12

**Authors:** Rong Li, Shanshan Wang, Ling Zhu, Jian Guo, Ling Zeng, Qiyong Gong, Li He, Huafu Chen

**Affiliations:** 1 Key Laboratory for Neuroinformation of Ministry of Education, School of Life Science and Technology, University of Electronic Science and Technology of China, Chengdu, PR China; 2 Department of Neurology, West China Hospital of Sichuan University, Chengdu, PR China; 3 Huaxi MR Research Center, Department of Radiology, West China Hospital of Sichuan University, Chengdu, PR China; Cuban Neuroscience Center, Cuba

## Abstract

**Background:**

Transient ischemic attack (TIA) is usually defined as a neurologic ischemic disorder without permanent cerebral infarction. Studies have showed that patients with TIA can have lasting cognitive functional impairment. Inherent brain activity in the resting state is spatially organized in a set of specific coherent patterns named resting state networks (RSNs), which epitomize the functional architecture of memory, language, attention, visual, auditory and somato-motor networks. Here, we aimed to detect differences in RSNs between TIA patients and healthy controls (HCs).

**Methods:**

Twenty one TIA patients suffered an ischemic event and 21 matched HCs were enrolled in the study. All subjects were investigated using cognitive tests, psychiatric tests and functional magnetic resonance imaging (fMRI). Independent component analysis (ICA) was adopted to acquire the eight brain RSNs. Then one-sample t-tests were calculated in each group to gather the spatial maps of each RSNs, followed by second level analysis to investigate statistical differences on RSNs between twenty one TIA patients and 21 controls. Furthermore, a correlation analysis was performed to explore the relationship between functional connectivity (FC) and cognitive and psychiatric scales in TIA group.

**Results:**

Compared with the controls, TIA patients exhibited both decreased and increased functional connectivity in default mode network (DMN) and self-referential network (SRN), and decreased functional connectivity in dorsal attention network (DAN), central-executive network (CEN), core network (CN), somato-motor network (SMN), visual network (VN) and auditory network (AN). There was no correlation between neuropsychological scores and functional connectivity in regions of RSNs.

**Conclusions:**

We observed selective impairments of RSN intrinsic FC in TIA patients, whose all eight RSNs had aberrant functional connectivity. These changes indicate that TIA is a disease with widely abnormal brain networks. Our results might put forward a novel way to look into neuro-pathophysiological mechanisms in TIA patients.

## Introduction

Transient ischemic attack (TIA) is usually defined as a neurologic ischemic disorder without permanent cerebral infarction and lasting neurological deficits [1]. Yet, several evidences have demonstrated that patients suffered a TIA can have lasting cognitive impairment, despite the recovery of focal neurological deficits [2]. In a meta-analysis of the risk of cerebral angiography in patients with TIA or stroke, the risk of transient neurological deficit was 3.0% and the risk of permanent neurological deficit was 0.7% [3]. There was a study reported that the deficits in executive function, information processing speed, visual memory, abstraction, and visuoconstruction were found in these patients, which may result from the disturbance of frontal functions [4]. And another research also found that TIA patients with carotid artery occlusion may develop persisting impairment in execution and reaction [5]. However, all these studies have been used the neuropsychological test scores to evaluate the cognitive abnormalities in TIA patients, which may relatively be insensitive to mild cognitive impairment and can not provide any information about the mechanism of such cognitive decline.

Recent neuroimaging studies suggested that widespread resting state brain networks were altered in many neurological and psychiatric disorders [6], [7]. Functional connectivity in resting state has became a prevalent technique to study the brain networks without specifically designed behavioral tasks. Functional connectivity represents the temporal synchronization of neuronal activities between different brain regions within a network, and can be used to reflect the normal or abnormal state of the corresponding functions [8], [9]. Besides, several related studies have demonstrated that inherent brain activity in the resting state is spatially organized in a set of specific coherent patterns [10], [11]. These patterns were named resting state networks (RSNs), which epitomize the functional architecture of memory networks, language, attention, visual, auditory and somato-motor [12]. Despite that there is not a consensus yet, a number of consistent RSNs, as detected by independent component analysis (ICA), could be jointly reported in the same study [10].

So far, it is unclear about the changes in the functional architecture of RSNs in TIA during resting state. Some prior studies found cognitive impairment in TIA patients on attention, memory, reaction and execution compared to healthy controls [4], [5]. It could be explained just in the light of abnormal regional responses, but they may also reflect normal responses within a dysfunctional network. In this case, researches on RSNs in TIA patients would provide useful data to confirm the network impairment hypothesis. In the present study, we evaluated the topological differences of the RSNs between TIA patients and healthy controls, using the method of ICA on fMRI data of resting state. We mainly aim to verify 1) whether the functional connectivity of any RSNs may be altered in TIA patients and 2) if so, whether these changes are related to the measured clinical scales. The results of this research may employ a new manner to investigate the neurological and neurophysiological mechanisms of TIA.

## Methods

### Subjects

The study protocol was approved by the institutional ethics committee at Sichuan University. Written informed consent was obtained before each subject’s participation in the trial. 21 TIAs suffered an ischemic event were enrolled in the study. A TIA was defined according to the World Health Organization recommendations as any syndrome of focal neurologic dysfunction ascribable to a vascular territory and lasted for less than 24 hours [13]. Those with brain lesions on fluid attenuated inversion recovery (FLAIR) images or T2-weighted images, leukoaraiosis or psychiatric diseases were excluded. The control subjects were healthy volunteers matched for age, sex, years of education, and with no history of stroke/TIA or other neurological disorders. All subjects had a complete blood count and metabolic profile testing, an ultrasonic cardiogram, a Holter monitor and a carotid duplex ultrasound examination.

### Cognitive and Psychiatric Assessment

Cognitive and psychiatric assessments of these subjects were conducted by two independent neuropsychologists. The Montreal Cognitive Assessment (MoCA) was used for assessing the general condition of cognitive function, which is a useful brief tool to assess cognition in TIA patients with mild cognitive impairment(MCI) [14]. The Auditory Verbal Memory Test (AVMT, Chinese version based on California Verbal Learning test) was for the verbal memory and the backward Digital Span Test (DST-backward) was for working memory. The Hamilton Anxiety (HARS) and Depression (HDRS) Rating Scales were also administered to rate the psychiatric characteristics in all subjects.

### MRI Acquisition

All TIA patients experienced symptoms with acute onset of paralysis or numbness which less than 1 month before the MRI examination. And the median duration of symptoms in TIA patients was 48 min (ranging from 15 min to 1.5 h).Imaging was performed on a 3 Tesla Trio scanner (Siemens AG). The resting-state fMRI was obtained by using an echo planar imaging sequence with following protocols: TR = 2000 ms, TE = 30 ms, field of view, 240×240 mm^2^, acquisition matrix, 64×64, and slice thickness, 5 mm, voxel size = 3.75×3.75×5 mm. This acquisition sequence generated 190 volumes in 6 min and 24 s. During the fMRI scanning, all subjects were informed to keep still with their eyes closed, to think of nothing in particular and remain awake. A 3D time-of-flight MR angiography (MRA) was performed to visualize the cerebral vasculature of the subjects and 3D high-resolution T1-, T2-weighted. High-resolution anatomical images of the whole brain were acquired with a volumetric three-dimensional spoiled gradient recall sequence to detect clinically silent lesions. (TR = 1900 ms, TE = 2.28 ms, 240×240 mm^2^, matrix, 256×256,whole head: 176 sagittal slice, slice thickness, 1.0 mm with no gap, voxel size = 0.9×0.9×1 mm).

### Data Processing and Statistical Analysis

#### Data preprocessing

Data preprocessing was carried out by using the SPM8 package (http://www.fil.ion.ucl.ac.uk/spm). The first ten volumes were discarded because of instability of the initial MRI signal. The remaining 180 volumes were first carried out slice timing correction and slice realignment for head motion correction. No subject’s translational or rotational parameters in a data set exceeded ±1 mm or ±1°. Therefore, no datasets were excluded from the analysis. The functional images were realigned with the corresponding T1-volume and warped into a standard stereotaxic space at a resolution of 3×3×3 mm^3^, using the Montreal Neurological Institute (MNI) echo-planar imaging template in SPM8. Then, they were spatially smoothed by convolution with an isotropic Gaussian kernel (FWHM = 8 mm).

#### ICA and identification of RSNs

Group spatial ICA was performed using the GIFT software (http://icatb.sourceforge.net/, version 1.3e) [15]. For the specific process, please see Methods S1.

#### Second-level analysis of the RSNs

The ICs in line with eight RSNs were extracted from all subjects. One-sample t-tests were then calculated in each group to gather the spatial maps of each RSNs. Thresholds were set at p<0.001 (FDR corrected) [16]. To compare the changes of the RSNs between TIA and HC, two-sample t-tests were calculated (p<0.001, FDR corrected). The group comparisons were masked to the voxels within corresponding RSNs. The mask was created by combining the regions of corresponding RSNs in both TIA and HC, which were obtained from one-sample t-tests results (p<0.001, FDR corrected) [17].

#### Correlation analyses

A Pearson correlation analysis was performed to explore the relationship between Z values in network maps and neuropsychological scores. The voxels in corresponding RSNs showing significantly different Z values between TIA and HC groups were extracted as a mask, which consists of several regions of interest (ROIs). These ROIs were applied to all subjects. The mean Z values of each individual within these ROIs were correlated to the neuropsychological scores and then thresholded at a significance level of p<0.05.

## Results

### Subjects’ Characteristics

Demographic profiles and risk factors of both groups are presented in Table 1. Patients with TIA showed only significant differences compared with normal controls in carotid artery stenosis (P = 0.006). The results of the cognitive and psychiatric tests are summarized in Table 2. The TIA patients tended to have poorer scores in MoCA, AVMT, and DST-backward but without statistical significant difference. There were no significant differences in HARS and HDRS between two groups.

**Table 1:** Demographic characteristics and risk factors of participants.

Characteristics	TIA Patients (n = 21)	HC (n = 21)	P value
Age, mean (SD)	50.1(6.5)	48.2(7.9)	0.39
Sex, males (%)	15(0.71)	13(0.62)	0.51
Education, mean years (SD)	10.4(2.1)	10.5(2.8)	0.95
Risk factors (%)			
Hypertension	8(0.38)	5(0.24)	0.32
Diabetes	3(0.14)	1(0.05)	0.29
Hyperlipidemia	7(0.33)	6(0.28)	0.74
Atrial Fibrillation	0	0	
Previous stroke	0	0	
Smoking	5(0.24)	2(0.09)	0.21
Carotid artery stenosis	11(0.52)	2(0.09)	**0.006**
Intracranial arteries stenosis	2(0.09)	0(0)	0.49

Abbreviations: TIA, Transient ischemic attack; HC, Healthy controls; SD, standard deviation.

**Table 2:** Cognitive and psychiatric tests of participants.

	TIA Patients (n = 21)	Controls (n = 21)	P value
MoCA, mean (SD)	24.5(3.5)	26.2(1.6)	0.053
AVMT, mean (SD)	47.9(15.8)	55.7(19.8)	0.065
DST-backward, mean (SD)	5.1(1.9)	5.7(3.4)	0.060
HARS, mean (SD)	9.3(2.7)	8.3(2.3)	0.224
HDRS, mean (SD)	6.5(2.1)	5.6(1.7)	0.142

Abbreviations: MoCA, Montreal Cognitive Assessment; AVMT, Auditory Verbal Memory Test; DST-backward, Backward Digital Span Test; HARS, Hamilton Anxiety Rating Scales; HDRS, Hamilton Depression Rating Scales; SD, standard deviation.

### Spatial Pattern of RSNs in each Group

**Figure 1:**
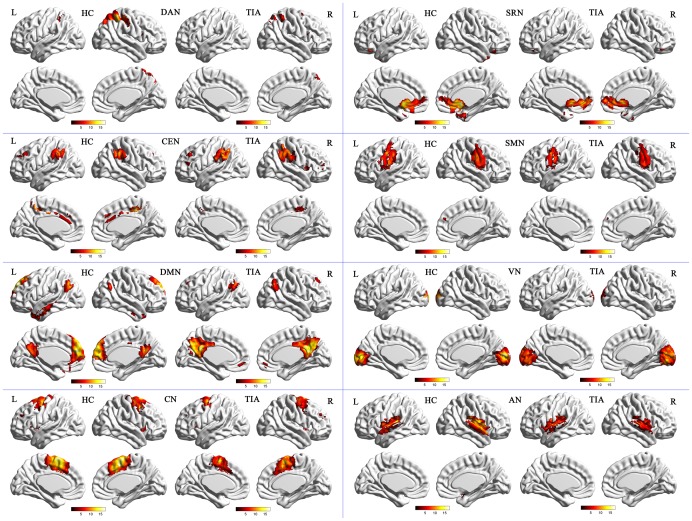
Cortical representation of the eight group-level RSNs in HC and in TIA groups. Lateral and medial views of left hemisphere and lateral and medial views of right hemisphere for each groups. The color scale represents T values in each RSN (maps thresholded at p<0.001, FDR corrected).

The one-sample t-tests revealed a typically spatial pattern in each RSN in both TIA and HC group. Our results for IC classification and spatial pattern were consistent with the results of previous studies [10], [18], which are illustrated in Fig. 1.

### Aberrant RSNs in Patients with TIA

**Figure 2:**
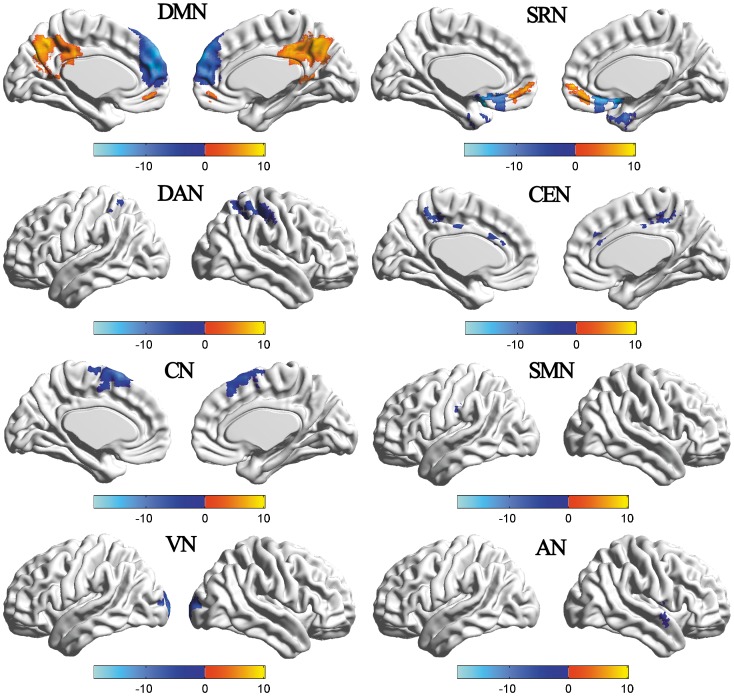
3D renders of a two-sample t-test each RSNs in the TIA vs. HC (p<0.001, FDR corrected). The warm and cold colors indicate the brain regions with significantly increased and decreased functional connectivity in TIA, respectively.

The two-sample t-tests revealed the differences in functional connectivity between the two groups (Fig. 2; Table 3). Compared to HC, TIA patients illustrated both increased and decreased (p<0.001, FDR corrected) functional connectivity in the DMN and SRN, and TIA patients exhibited specifically decreased (p<0.001, FDR corrected) functional connectivity in the DAN, CEN, CN, SMN, VN and the AN(clustering extent threshold, 15 voxels). Table 3 summarizes for the abnormal functional connectivity brain regions of each RSN.

**Table 3:** The differences in functional connectivity between the two groups.

RSNs	Brain areas	MNI			*T* values	Voxel size
		X	Y	Z		
DMN	L-Middle Temporal Gyrus	−63	−15	−18	−12.5921	30
	L-Inferior Temporal Gyrus	−48	3	−36	−8.0991	30
	R-Superior Frontal Gyrus	12	57	24	−18.8929	638
	L-Medial Frontal Gyrus	−3	51	−9	6.4498	27
	L-Cingulate Gyrus/Precuneus	−9	−69	36	10.2085	705
	R-Angular Gyrus	42	−63	51	5.7933	29
SRN	L-Medial Prefrontal Gyrus	−39	54	−9	−19.8424	86
	L-Superior Frontal Gyrus	0	51	−6	8.4107	34
DAN	R-Inferior Parietal Lobule	42	−36	60	−7.9824	91
	L-Superior Parietal Lobule	−36	−54	63	−6.3067	12
CEN	R-Precuneus	9	−24	39	−10.9888	89
	R-Anterior Cingulate	3	33	27	−6.6113	40
CN	L-Superior Frontal Gyrus	−3	15	63	−11.5482	356
	R-Medial Frontal Gyrus	−33	−15	51	−6.8303	36
SMN	L-Postcentral Gyrus	−48	−18	30	−6.4079	32
VN	R-Middle Occipital Gyrus	24	−96	−3	−10.6933	63
	L-Middle Occipital Gyrus	−3	−105	−3	−14.1585	46
AN	R-Superior Temporal Gyrus	51	−3	12	−6.2424	39

Abbreviations: RSNs, Resting state networks; L, Left; R, Right; FC, Functional connectivity; TIAs, Transient ischemic attack patients; HC, Healthy controls; MNI, Montreal Neurologic Institute; DMN, default mode network; SRN, self-referential network; DAN, dorsal attention network; CEN, central-executive network; CN, core network; SMN, somato-motor network; VN, visual network; AN, auditory network. All the coordinates are donated by Montreal Neurological Institute (MNI) space coordinates. T score represents the statistical value of peak voxel showing the differences in functional connectivity between the two groups. Positive and negative T values indicate increased and decreased functional connectivity in the TIA group, respectively.

However, no significant correlation was found between brain regions with aberrant functional connectivity and neuropsychological scores.

## Discussion

In the present study, we mainly focused on the confirmation of whether the intrinsic functional connectivity of RSNs was altered or not in patients with TIA and on detecting whether there was a link between these changes and the disease severity.

Previous studies have demonstrated that the most common cause of TIA is atherosclerosis, which usually occurs in one of the internal carotid arteries [19]. Meanwhile, some other studies have suggested that the occlusion of internal carotid arteries may be a major risk factor for cognitive impairment [20], [21]. It’s reported that individuals who experienced a TIA have some changes in cognitive functions, such as attention, working memory, and learning and memory, which rely on DAN, SRN, CN and DMN. We also chose other RSNs (including VN, AN, SMN and CEN) in our study. Different regions belonging to these networks have already showed abnormal brain activations in TIA patients as compared with HC. We found that functional connectivity was significantly different among eight RSNs. Both increased and decreased functional connectivity was found in the DMN and the SRN and decreased functional connectivity was found in all other networks in TIA patients. These results were similar to the literature on differential activations of specific cerebral regions in nondisabling transient ischemic attacks (TIA) and minor strokes patients as compared with healthy controls.

The default mode network (DMN) is engaged in maintaining the baseline brain activities associated with cognitions of pisodic memory, and environmental monitoring [22]. As we know, DMN exhibited high levels of activity during resting state and decreased the activity for processes of externally oriented mental activity, which was induced by a wide range of sensory and cognitive tasks [23]. Our findings indicated that the left middle temporal gyrus, inferior temporal gyrus and right superior frontal gyrus showed decreased functional connectivity in TIA patients as compared with HC. Previous functional neuroimaging studies have suggested that the middle temporal gyrus and inferior temporal gyrus are involved in several cognitive processes, including language and semantic memory processing, as well as visual perception [24], [25]. Furthermore, the superior frontal gyrus (SFG) is thought to contribute to higher cognitive functions and particularly to working memory [26], [27]. Together, these may suggest that the dysfunction of middle temporal gyrus and superior frontal gyrus could be resulted from long-term existence of various ischemic risks and was related to the impairment of cognitive function of TIA patients.

In the present study, the aberrant functional connectivity of DMN in TIA patients was also represented by the increased functional connectivity in the left medial frontal gyrus, left posterior cingulate/precuneus and right angular gyrus. Recent neuroimaging studies have shown that the posterior cingulate cortex (PCC), ventral medial prefrontal cortex (vmPFC) and the bilateral angular gyrus were more active during resting state than during cognitive tasks [28], [29]. In addition, the angular gyrus and posterior cingulate/precuneus were significantly activated during memory retrieval [30]. What’s more, the mPFC is supposed to provide information from prior experiences in the form of memories during the construction of self-relevant mental simulation. These findings have suggested that medial prefrontal cortex played an important role in the prominent cognitive behavioral models of TIA. It has been showed that DMN played a critical role in social cognition and particularly in the relationship between the self and the social environment in TIA patients.

The SRN has already shown special physiological characteristics with a high level of neural activity during resting conditions [18], [31]. In this study, patients with TIA showed significant decreased functional connectivity in left medial prefrontal cortex, and increased functional connectivity in left superior frontal gyrus in SRN compared to HC. There were several studies indicating that monitoring-related medial prefrontal cortex activity was served as a signal that was engaged regulatory processes in the lateral prefrontal cortex to implement performance adjustments [32]. Thus, it may hint that the reduction of functional connectivity of the medial prefrontal cortex in SRN may affect the self-related activity of TIA patients. In fMRI experiments, Goldberg et al. have found evidence that the superior frontal gyrus is involved in self-awareness, in coordination with the action of the sensory system [33]. According to the definition of TIA, the symptoms of a TIA are short-lived and usually last a few seconds to a few minutes and most symptoms disappear within 60 minutes [1]. This may indicate that the increase of functional connectivity of the superior frontal gyrus regulated the recovery process of TIA patients after onset of illness.

DAN is thought to mediate goal-directed top-down processing. It is also involved in many higher-order cognitive tasks [34]. Disruption of interhemispheric FC was reported to significantly correlate with abnormal detection of visual stimuli in the attention network in stroke patients [35], who share the similar pathophysiology with TIAs. We found that the right inferior parietal lobule (IPL) and left superior parietal lobule presented the decreased functional connectivity within DAN in TIA patients. We reviewed evidences which showed that the right IPL played an important role in two different aspects of attention: maintaining attentive control on current task goals [36], [37] as well as responding to salient new information or alerting stimuli in the environment [38], [39]. Therefore, damage to the right IPL may lead to deficits in both maintaining attention and responding to salient events in TIA patients. Previous study suggested that the superior parietal lobe was critical for sensorimotor integration by maintaining an internal representation of the body’s state [40]. Our findings may reflect that the two brain regions within DAN play a role in cognitive regulation and functional maintenance in TIA patients.

A central-executive network (CEN) is responsible for high-level cognitive functions [41]. The CEN plays a critical role in the active maintenance and manipulation of information in working memory, and in judgment and decision making in the context of goal directed behavior [42].We knew that TIA patients may develop persisting impairment in execution, visual attention function and working memory. However, the relation between brain regions belonging to CEN and behavioral expression of TIA patients need to be further studied. Besides, our study found the decreased functional connectivity of the anterior cingulate cortex in CEN in TIA patients compared with HC. It has been suggested that the top-down attention control is mediated by the anterior cingulate cortex (ACC) [43], [44]. The ACC is connected with the prefrontal cortex and parietal cortex as well as the motor system and the frontal eye fields [45], which makes it a central station for processing top-down and bottom-up stimuli and assigning appropriate control to other areas in the brain [46]. To sum up, these findings may thus reflect the functional impairment of CEN in the cognitive control of TIA.

Some research found that core network (CN) was not only associated with task control function [47], [48], but also involved in “salience” processing during resting state [49]. Another important role of CN is to switch between the DMN and task-related networks in cognitive control [50]. We found decreased functional connectivity in left superior frontal gyrus and right medial frontal gyrus. Previous researches have suggested that the superior frontal gyrus and medial frontal gyrus may form part of an attention or executive control system [47], [51], and TIA patients also have the impairment in these functions. This implied that these brain regions within CN played a significant role in cognitive control of TIA patients.

In general, the perceptual systems consisted of the visual, auditory and somato-motor networks, which can be considered at the lower-order of the cognitive processing hierarchy. Our results indicated that TIA patients presented a certain decreased functional connectivity among SMN (left postcentral gyrus), VN (bilateral middle occipital gyrus) and AN (right superior temporal gyrus) compared with HC. What’s more, some previous researches have demonstrated the functions of these brain regions: the lateral postcentral gyrus was involved in somatosensory processing from the heterolateral side of the body [52], [53]; the middle occipital gyrus was involved in visual function [54], [55], memory [56], [57], language [58], [59] and some other functions [60], [61]; the superior temporal gyrus was involved in auditory processing, but also has been implicated as a critical structure in social cognition [62]. Furthermore, known from clinical history and the previous studies, the most frequent symptoms of TIA patients include temporary loss of vision, difficulty speaking, weakness on one side of the body, and numbness or tingling, usually on one side of the body [63]. Therefore, the aberrant functional connectivity of SMN, VN and AN found in our study may be associated with perceptual impairments in TIA patients.

Unfortunately, the study failed to demonstrate significant correlation between RSNs and neuropsychological scores. The possible reason might be that the patients we recruited were young and only had a short disease history. Therefore, function alteration may not be significantly enough to be explored by traditional psychological scales used in our study. Actually, this was in line with the hypothesis that there was a long presymptomatic period in the development of cognitive impairment and psychiatric disturbances after the ischemic attack [64].

Several limitations of the current study are deserved to be mentioned. First, the sample size for TIA patients is small, with variations in the age and disease on-set time, which might affect the statistical analysis and results of this study. In addition, the ICA method is not able to provide information on the functional connectivity in all brain regions, for instance, the limbic system [65]. Furthermore, the neurophysiological meaning of the RSNs still remains unclear. Finally, although a lot of literature revealed that spontaneous brain activity [66] is organized into RSNs [18], [67], so far, the RSNs hasn’t provide a complete description of brain functional architecture.

### Conclusions

In conclusion, we observed selective impairments of RSN intrinsic FC in TIA patients, whose all eight RSNs had aberrant functional connectivity. Both increased and decreased functional connectivity was found in the DMN and SRN, and decreased functional connectivity was exhibited in all other RSNs in TIA patients compared to HC. Our fMRI study might potentially put forward a novel way to understand the neuro-pathophysiological mechanism of cognition function changes in TIA.

## Supporting Information

Methods S1 **Supporting methods.**
(DOC)
