# Supplementary material for: Aberrant Functional Connectivity of Resting State Networks in Transient Ischemic Attack
Source: PLoS One. 2013 Aug 12;8(8):e71009. doi: 10.1371/journal.pone.0071009 (PMC3741391; doi:10.1371/journal.pone.0071009)
Supplement: Methods S1 — Supporting methods. (DOC) [file pone.0071009.s001.doc]

**Supplementary Material**

**Method**

This involves a preliminary dimension estimation on all subjects to determine the number of independent components (ICs), using the minimum description length (MDL) criterion . Then, fMRI datas of all subjects in each group were concatenated and the temporal dimension of the whole data set was reduced by means of principal component analysis (PCA), respectively, followed by an IC estimation using the FastICA algorithm . Two separate groups of spatial ICA were both conducted on the TIA and HC groups, with 46 and 34 ICs respectively, which ensured that the RSNs had similar spatial pattern in the two groups . IC time-courses and spatial maps for each subject were reconstructed, using the aggregated components and the results from the data reduction step . For each IC, the time-course corresponds to the waveform of a specific pattern of coherent brain activity, and the intensity of this pattern of brain activity across the voxels was expressed in the associated spatial map . In order to display the voxels that contributed most strongly to a particular IC, the intensity values in each spatial map were converted to Z values, removing the average value and being divided by the standard deviation of the intensity distribution . Because ICA on fMRI data extracts patterns of coherent neuronal activity (i.e. networks), it is commonly accepted that Z values can indirectly provide a measure of functional connectivity within the network .

An implement was in the GIFT software , the components to be retained for further analysis among the 46/34 estimated ICs for two groups were selected based on the largest spatial correlation with specific RSN templates from our previous studies , besides, these templates have also been used in another resting state fMRI study , in combination with the spatial correlation for the RSN selection criterion. The eight RSN templates from our previous studies used in the current work were: dorsal attention network (DAN; central-executive network (CEN); default mode network (DMN); core network (CN); self-referential network (SRN); somato-motor network (SMN); visual network (VN) and auditory network (AN).

**Reference**

1. Jafri MJ, Pearlson GD, Stevens M, Calhoun VD (2008) A method for functional network connectivity among spatially independent resting-state components in schizophrenia. NeuroImage 39: 1666-1681.

2. Li YO, Adali T, Calhoun VD (2007) Estimating the number of independent components for functional magnetic resonance imaging data. Hum Brain Mapp 28: 1251-1266.

3. Liao W, Chen H, Feng Y, Mantini D, Gentili C, et al. (2010) Selective aberrant functional connectivity of resting state networks in social anxiety disorder. NeuroImage 52: 1549-1558.

4. Liao W, Mantini D, Zhang Z, Pan Z, Ding J, et al. (2010) Evaluating the effective connectivity of resting state networks using conditional Granger causality. Biol Cybern 102: 57-69.

5. Hyvarinen A (1999) Fast and robust fixed-point algorithms for independent component analysis. IEEE Trans Neural Netw 10: 626-634.

6. Calhoun VD, Adali T, Pearlson GD, Pekar JJ (2001) A method for making group inferences from functional MRI data using independent component analysis. Hum Brain Mapp 14: 140-151.

7. Mantini D, Perrucci MG, Del Gratta C, Romani GL, Corbetta M (2007) Electrophysiological signatures of resting state networks in the human brain. Proc Natl Acad Sci U S A 104: 13170-13175.

8. Bartels A, Zeki S (2005) Brain dynamics during natural viewing conditions--a new guide for mapping connectivity in vivo. NeuroImage 24: 339-349.

9. Beckmann CF, DeLuca M, Devlin JT, Smith SM (2005) Investigations into resting-state connectivity using independent component analysis. Philos Trans R Soc Lond B Biol Sci 360: 1001-1013.

10. Damoiseaux JS, Rombouts SA, Barkhof F, Scheltens P, Stam CJ, et al. (2006) Consistent resting-state networks across healthy subjects. Proc Natl Acad Sci U S A 103: 13848-13853.

11. Greicius MD, Flores BH, Menon V, Glover GH, Solvason HB, et al. (2007) Resting-state functional connectivity in major depression: abnormally increased contributions from subgenual cingulate cortex and thalamus. Biol Psychiatry 62: 429-437.

12. van de Ven VG, Formisano E, Prvulovic D, Roeder CH, Linden DE (2004) Functional connectivity as revealed by spatial independent component analysis of fMRI measurements during rest. Hum Brain Mapp 22: 165-178.

13. van de Ven V, Bledowski C, Prvulovic D, Goebel R, Formisano E, et al. (2008) Visual target modulation of functional connectivity networks revealed by self-organizing group ICA. Hum Brain Mapp 29: 1450-1461.

14. Mantini D, Corbetta M, Perrucci MG, Romani GL, Del Gratta C (2009) Large-scale brain networks account for sustained and transient activity during target detection. NeuroImage 44: 265-274.
